# Simultaneous Multiclass Analysis of Cyanotoxins in Cyanobacterial Samples Using Hydrophilic Interaction Liquid Chromatography‐Tandem Mass Spectrometry

**DOI:** 10.1002/jssc.70121

**Published:** 2025-03-19

**Authors:** Rosemary Bergin, Siobhan Peters, Simon Mitrovic, David P. Bishop

**Affiliations:** ^1^ Hyphenated Mass Spectrometry Laboratory School of Mathematical and Physical Sciences University of Technology Sydney Ultimo New South Wales Australia; ^2^ School of Life Sciences University of Technology Sydney Ultimo New South Wales Australia

**Keywords:** algal blooms, cyanobacteria, cyanotoxins, hydrophilic interaction liquid chromatography, multiclass analysis

## Abstract

The proliferation of cyanobacteria can result in algal blooms, which may cause environmental and biological harm due to the production and release of secondary metabolites, or cyanotoxins, into the affected waterway. Cyanobacteria can produce multiple classes of cyanotoxins; therefore, to understand the full toxic load of algal blooms, it is necessary to perform analyses that quantify each class. These classes are generally monitored individually due to the challenges associated with the differing physicochemical properties of the cyanotoxins. Hydrophilic interaction liquid chromatography (HILIC) is a form of chromatography capable of retaining multiple classes of cyanotoxins that differ in physicochemical properties. Here an HILIC‐MS/MS method was developed and validated to detect 3 microcystins, 11 saxitoxins, and 2 anatoxins. The chromatographic conditions were optimized to allow for the separation of multiple pairs of saxitoxin epimers, and in‐source fragmentation in the MS interface was used to develop unique MRMs between the pairs. The method was validated and had low limits of detection (LODs, between 0.00770 and 9.75 µg L^−1^) and limits of quantification (LOQs, between 0.0257 and 32.5 µg L^−1^) for all compounds. All analytes exhibited good linearity (*R* ^2^ values ≥ 0.991) and low percentage relative standard deviations for retention time (0%–1.74%) and peak area (4.54%–27.6%), with spiked recoveries ranging from 75.6% to 117% for all compounds. A multiclass sample preparation method to extract the three classes of analytes from cyanobacterial samples was developed and validated, with 80:20 acetonitrile:water and 0.1% formic acid as the optimal extraction solvent. The newly developed sample preparation and analysis methods were applied to cultured cyanobacteria and field samples, with microcystins and saxitoxins detected. The multiclass sample preparation and analysis methods developed here improve on individual methods as they reduce the complexity and time of sample preparation and analysis and will assist ecotoxicologists in assessing the full toxic risk of cyanobacterial blooms.

## 1 Introduction

Cyanobacteria are ancient, prokaryotic, photosynthetic phytoplankton found predominantly in water, but can also live in soil, snow, and rocks [1, 2, 3]. Cyanobacteria play a crucial role in the environment as a biological pump by consuming carbon dioxide, helping to maintain atmospheric carbon dioxide levels [4]. When cyanobacteria grow to large biomass, termed an “algal bloom,” they become visible as a green color, and if buoyant, may float to the water surfaces, making unsightly scums. The latter allows for a growth advantage due to increased availability of light and carbon dioxide [1, 2, 4, 5]. Algal blooms negatively impact aquatic ecosystems by decreasing the amount of dissolved oxygen in water bodies, resulting in hypoxic or anoxic conditions that may harm aquatic life [6]. Blooms also decrease water quality, altering the color, odor, and taste due to the production of geosmin and 2‐methylisoborneol [7].

Some cyanobacteria can produce toxic secondary metabolites, known as cyanotoxins [2]. The release of cyanotoxins from algal blooms into the surrounding environment occurs through maturation, but particularly after cell death [8]. Cyanotoxins have been associated with numerous animal and human poisonings worldwide [9]. Poisoning can occur through recreational activities such as swimming and bathing, but more commonly occurs through drinking contaminated water. Drinking water is the most regulated and monitored route of exposure due to it having the potential to directly affect a relatively large number of people, causing 80% of human exposures to cyanotoxins [9, 10].

Each class of cyanotoxin has different chemical properties and toxicities. The most frequently observed and potent cyanotoxins are primarily alkaloids and cyclic peptides, with one ubiquitous group of toxic cyclic peptides, microcystins (MCs), being identified worldwide and responsible for multiple human and animal mortality events [11, 12]. There are over 279 analogues of MCs currently identified [13, 14], with the three most common analogues, MC‐RR, MC‐LR, and MC‐YR, being the subject of regulatory overview [15, 16]. Neurotoxic saxitoxins (STXs) and anatoxins (ATXs) are classes of cyanotoxins that are of increasing interest, with the occurrence of neurotoxin‐producing cyanobacteria a phenomenon in almost all geographical regions worldwide [17, 18]. ATXs are small, bicyclic secondary amines [19]. No human intoxication events have been reported from ATXs; however, many animal fatalities have been reported, including cattle, dogs, and birds [18]. STXs are produced from cyanobacteria, marine dinoflagellates, several types of invertebrates, and select fish species [10, 20]. STXs have been identified as the causative agent of numerous human and animal intoxications and fatalities, with cases documented as far back as 1927 [10], and an extensive *Anabaena* bloom event in the Darling River, Australia, leading to the death of approximately 1600 cattle and sheep in 1990 [21].

Many species of cyanobacteria produce multiple classes of toxins. Therefore, to fully assess the toxic load and the potential health impacts of a cyanobacterial bloom, it is necessary to determine all cyanotoxin classes present. There are multiple methods for analyzing cyanotoxins [22], with liquid chromatography‐tandem mass spectrometry (LC‐MS/MS) commonly used due to its high sensitivity and specificity. However, each class is usually analyzed separately due to their differing physicochemical properties requiring unique chromatographic conditions, resulting in multiple time‐consuming analyses. This has resulted in an increase in attempts at multiclass separation methods to streamline the identification of multiple cyanotoxins in a single analysis. Reverse‐phase liquid chromatography (RPLC) is the most commonly used method to separate cyanotoxins and has been employed to successfully analyze MCs and ATXs in a multiclass analysis [23, 24]. STX, due to their high polarity, are usually analyzed by hydrophilic interaction liquid chromatography (HILIC) [20, 25].

Validated multiclass methods that analyze a range of MCs, ATXs, and STXs are not currently available. This analysis presents a number of unique challenges, such as separating and resolving multiple cyanotoxin analogues within the same class, and the differing chemical properties of the classes that produce unique retention characteristics and their resultant affinities to chromatographic separation mechanisms. The aim of this study was to develop a sample preparation method and an HILIC‐MS/MS analysis to simultaneously detect STXs, ATXs, and MCs from cyanobacteria samples. The validated method was then applied for the multiclass analysis of a variety of field and cultured algal samples.

## 2 Experimental

### 2.1 Chemicals

Certified reference standards of gonyautoxins 1–5 (GTX 1 (33.8 mg L^−1^), GTX 2 (24.8 mg L^−1^), GTX 3 (8 mg L^−1^), GTX 4 (8 mg L^−1^), GTX 5 (20.2 mg L^−1^), N‐sulfocarbamoyl gonyautoxin‐2 and 3 (C1 (40.1 mg L^−1^) and C2 (11.5 mg L^−1^)), saxitoxin (STX (20.3 mg L^−1^)), decarbamoylsaxitoxin (dcSTX (18.7 mg L^−1^)), and decarbamoylgonyautoxin‐2 and 3 (dcGTX2 (40.1 mg L^−1^) and dcGTX3 (11.5 mg L^−1^)) were purchased from Cifga Laboratoria S.A. (Lugo, Spain). HPLC‐grade MC standard mix (RR (4.99 mg L^−1^), YR (5.14 mg L^−1^), and LR (5.12 mg L^−1^)) was purchased from Merck (Castle Hill, NSW, Australia), and ATX‐a (ANA‐a (10 mg L^−1^)), homoanatoxin‐a (HATX (4 mg L^−1^)), and C^13^ ANA‐a (10 mg L^−1^) were purchased from Gold Standard Diagnostics (Warminster, PA, United States). LC‐MS grade acetonitrile (ACN) and formic acid (FA) were purchased from Chem Supply (Gillman, SA, Australia), and ammonium formate (99% purity) from Merck (Castle Hill, NSW, Australia). Ultrapure water (18.2 MΩ cm) was obtained from a Sartorius ultra‐pure water purification system (Göttingen, Germany), 0.22 µm cellulose acetate Spin‐X centrifuge tube filters from Corning (Mulgrave, VIC, Australia); and Whatman glass microfiber paper 7.0 cm from Merck (Castle Hill, NSW, Australia) was used in the algal sample preparation process.

### 2.2 Extraction Procedure

Cultivated cyanobacterial and field bloom samples were centrifuged and filtered (Whatman glass microfiber paper, 7.0 cm) before three sequential freeze‐thaw cycles at −80°C to lyse the cells. Samples were then freeze‐dried. Approximately 10–50 mg of dry cyanobacteria mass was added to a spin filter, along with 400 µL of 80:20 ACN:H_2_O with 0.1% FA as an extraction solvent. The sample was sonicated for 20 min and centrifuged at 5000 rpm for 15 min at 17°C. The filtrate was collected and stored in the 4°C refrigerator until analysis, where it was spiked with internal standard (IS) and analyzed using LC‐MS/MS.

### 2.3 Instrumentation

HILIC‐MS/MS was conducted on an Agilent 1290 Infinity II LC coupled to an Agilent 6490 triple quadrupole mass spectrometer (Mulgrave, VIC, Australia) with a Waters BEH Amide column (2.1 × 100 mm, 1.7 µm particle size). Data analysis was performed on the Agilent MassHunter Qualitative and Quantitative Analysis Software. The flow rate was set to 0.5 mL min^−1^, with a column oven temperature of 40°C, and a 1 µL injection volume. Gradient elution was performed with mobile phase A consisting of 2 mM ammonium formate in ultrapure water at a pH of 3.5, and mobile phase B consisting of ACN + 0.25% FA (v/v). The mobile phase gradient commenced with B at 90% (0.00 min), which was held for 3.00 min; at 3.01 min, B was changed to 80%, 77% at 4 min, 73% at 5.5 min, and 70% at 8.99 min, before a 1 min hold at 50% from 9 to 10 min, at which point B returned to its initial composition and equilibrated for 15 min to address and mitigate any potential drift that could occur with repeat injections (Table S1). Time segmentation was used for each class of cyanotoxins (MRMs listed in Table S2), and the autosampler tray was chilled to 4°C. The MS/MS was run in positive mode with an ESI source with the following parameters: 175°C gas temperature, 250°C sheath gas temperature, 3500 V capillary voltage, gas flow of 14 L min^−1^, and a sheath gas flow of 11 L min^−1^.

### 2.4 Method Validation

A seven‐point calibration curve was used to validate the multiclass method, with mixed standards prepared at nominal concentrations of 0.1, 1, 10, 50, 100, 200, and 400 µg L^−1^ (concentrations varied dependent on the stock, see Section 2.1), where HATX and ANA‐a were normalized to C^13^ ANA‐a, using internal calibration and all MCs and STXs using external calibration. The 100 µg L^−1^ standard was used to determine the MRM parameters and source optimization. The calibration curve was repeated twice with a 1/*x* weighted calibration curve constructed for each analyte, 12 days apart in order to determine the intra‐ and inter‐day variability, with repeatability (%RSD) determined from seven repeat injections from one point of the standard curve (concentrations are in Table S3). The LOD was calculated as 3× the signal‐to‐noise ratio (S/N), and the LOQ was calculated as 10× the S/N. The accuracy of the LC‐MS/MS method was determined through spiking an extracted sample and determining the percentage matrix spike recovery (matrix spike concentrations are listed in Table S3).

## 3 Results and Discussion

### 3.1 Selection of Multiple Reaction Monitoring Transitions for STX Epimers

**FIGURE 1:**
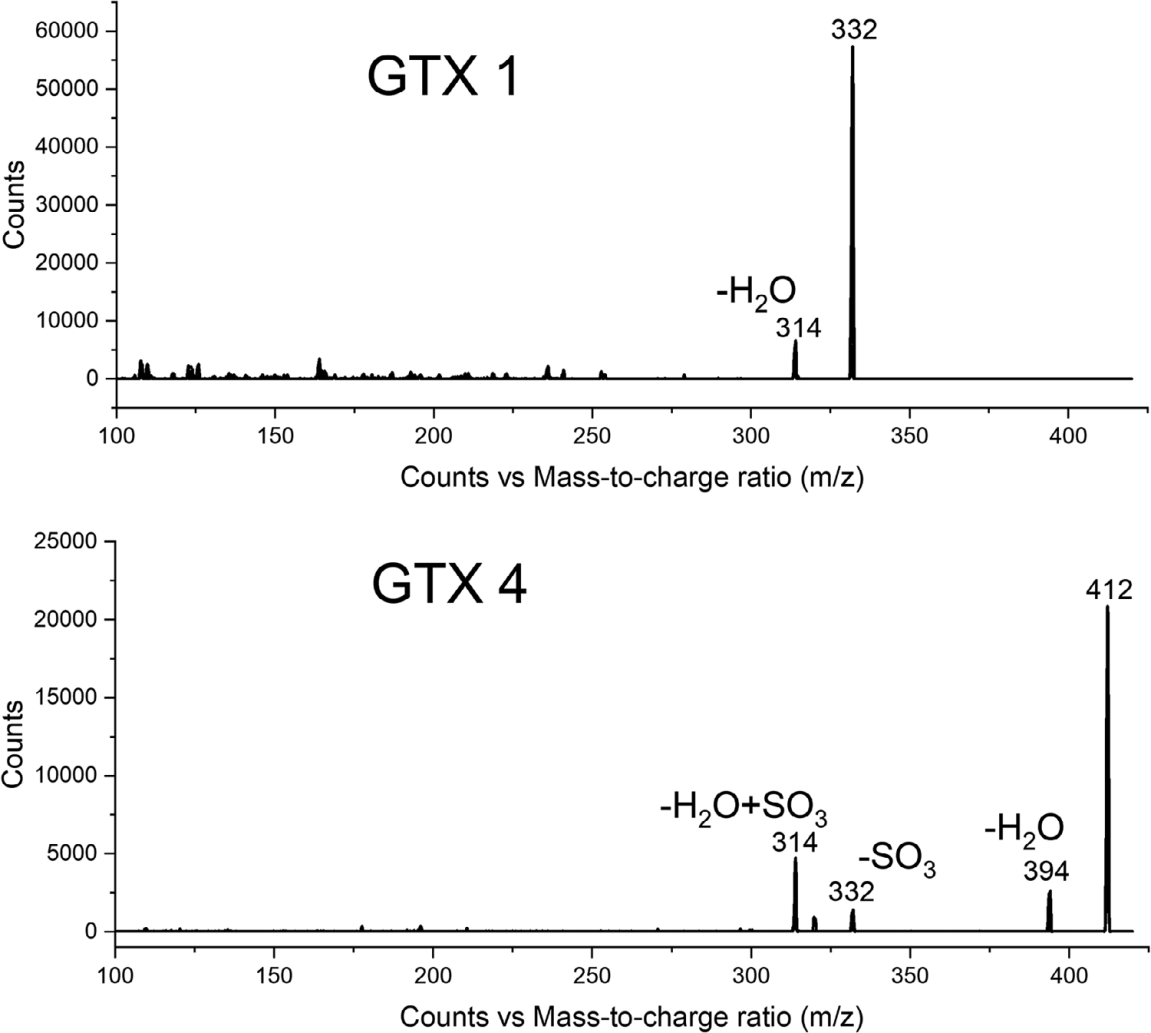
Precursor ion scans of GTX1 and GTX4, where the top pane is GTX 1 and the bottom pane is GTX 4, displaying their fragmentation patterns.

Each pair of STX epimers was supplied as a single solution, and during optimization of the MRM transitions, the precursor ion scans revealed a highly abundant ion for one of the pair that was not the intact protonated mass of the analyte. This aligned with the literature, which described that STX epimers can undergo in‐source fragmentation dependent on their stereochemical orientation [20, 26, 27]. An epimer is defined as a pair of stereochemical isomers that differ in absolute configuration around a chiral center [28, 29]. Without the in‐source fragmentation, selecting unique MRMs for these epimer pairs would be challenging, as they have identical masses and fragmentation patterns. This is exemplified in the MRM transitions for the epimer pair, GTX 1 and GTX 4, shown in Figure 1. Their protonated ion has an *m/z* of 412, and the quantifier ion for both compounds is 314 *m/z*. However, GTX 1 undergoes in‐source fragmentation and loses an SO_3_ group, and therefore has a precursor ion of 332 *m/z*. This allowed unique transitions (412 > 314 vs. 332 > 314, see Table S2) to be determined for this epimer pair. The precursor ion scans for the other epimer pairs are shown in Figures S2–S4.

The STXs that undergo in‐source fragmentation are defined by their stereochemical orientation of OSO_3_, i.e., whether it is in the R2 and R3 positions (β or α stereochemical orientation, respectively; see Figure S1 and Table S4). The epimer with an OSO_3_ in the α position preferentially undergoes in‐source fragmentation compared to the β isomer, which produces the molecular ion [30]. The elimination of the SO_3_ group is one of two dissociation products that can occur during ionization. When the analyte is protonated, it becomes metastable, allowing for the dissociation reaction to occur. The reaction involves either the neutral elimination of H_2_O, the cleavage of the S─O bond leading to the elimination of the SO_3_, or both eliminations occurring simultaneously [30]. The neutral H_2_O elimination can occur with all STXs analogues included within the method due to the presence of the hydroxyl group on Carbon‐12. The C‐12 hydroxyl group acts as a bridge between the geminal hydrogen of the guanidinium group and the hydroxysulfate group on Carbon‐11, allowing for a double proton transfer. The next step involves the migration of a proton from the hydroxysulfate group to the hydroxyl group, with the simultaneous dissociation of the C─O bond and the elimination of the neutral H_2_O [30, 31]. The SO_3_ elimination occurs when, as aforementioned, the analyte becomes metastable due to protonation. The C‐11α‐hydroxysulfate group is near the charge‐carrying protonated guanidine group, due to the positive charge being distributed across the guanidinium side of the molecule due to resonance. The protonated guanidinium then acts as an acid, withdrawing electrons from surrounding atoms, making the geminal hydrogen acidic. This acidic hydrogen catalyzes the breaking of the S─O bond, which is followed by the elimination of SO_3_ [30, 32].

It should be noted that the N‐sulfocarbamoyl toxins (C1, C2) lose two SO_3_ groups during ionization, resulting in their detection in the GTX 2 and GTX 3 MRM windows. This observation did not impact the analysis of the analytes because they were chromatographically resolved and identified with individual standards. This phenomenon could also potentially occur for analytes such as GTX 5 and STX, due to GTX 5 being a part of the N‐sulfocarbamoyl subclass; however, this was not observed in this study.

To harness this phenomenon for practical analysis, the method was optimized to favor SO_3_ elimination from the C‐11‐α‐stereoisomer while minimizing other potential in‐source fragmentation pathways such as the neutral loss of H_2_O. This optimization involved adjustments in gas flows, temperatures, and nebulizer voltages, resulting in differentiation between the stereoisomers while still allowing for maximum ionization of species that were not impacted, such as the other STXs, the ATXs, and the MCs.

### 3.2 Optimization of Multiclass Chromatographic Separation

The chromatographic method was optimized for the simultaneous analysis of three distinct classes of cyanotoxins, with each class requiring specific method adjustments to achieve the best possible separation and resolution. An HILIC column was employed as it had superior retention of polar analytes, with initial tests on the ACQUITY BEH amide HILIC column showing retention of all classes and separation of the distinct classes from each other. Previous attempts to use HILIC‐MS for the analysis of MCs found unsatisfactory peak shapes with increased broadening and tailing in comparison to the other toxins [26]. This was not seen here, and the column was selected for further optimization of the chromatographic conditions.

Optimization of the mobile phase included determining the optimal concentration of ammonium formate and the pH of the aqueous mobile phase, and the amount of FA added in the organic mobile phase. It should be noted that altering these factors did not impact the MCs and the ATXs, which exhibited quite robust retention; however, the focus was on the greater number of STXs and the epimer pairs. Baseline separation is not always required with LC‐MS/MS due to the increased selectivity of the MRMs; however, it was required here to further differentiate the epimer pairs, which, despite the in‐source fragmentation resulting in one unique MRM, still had shared MRM transitions.

**FIGURE 2:**
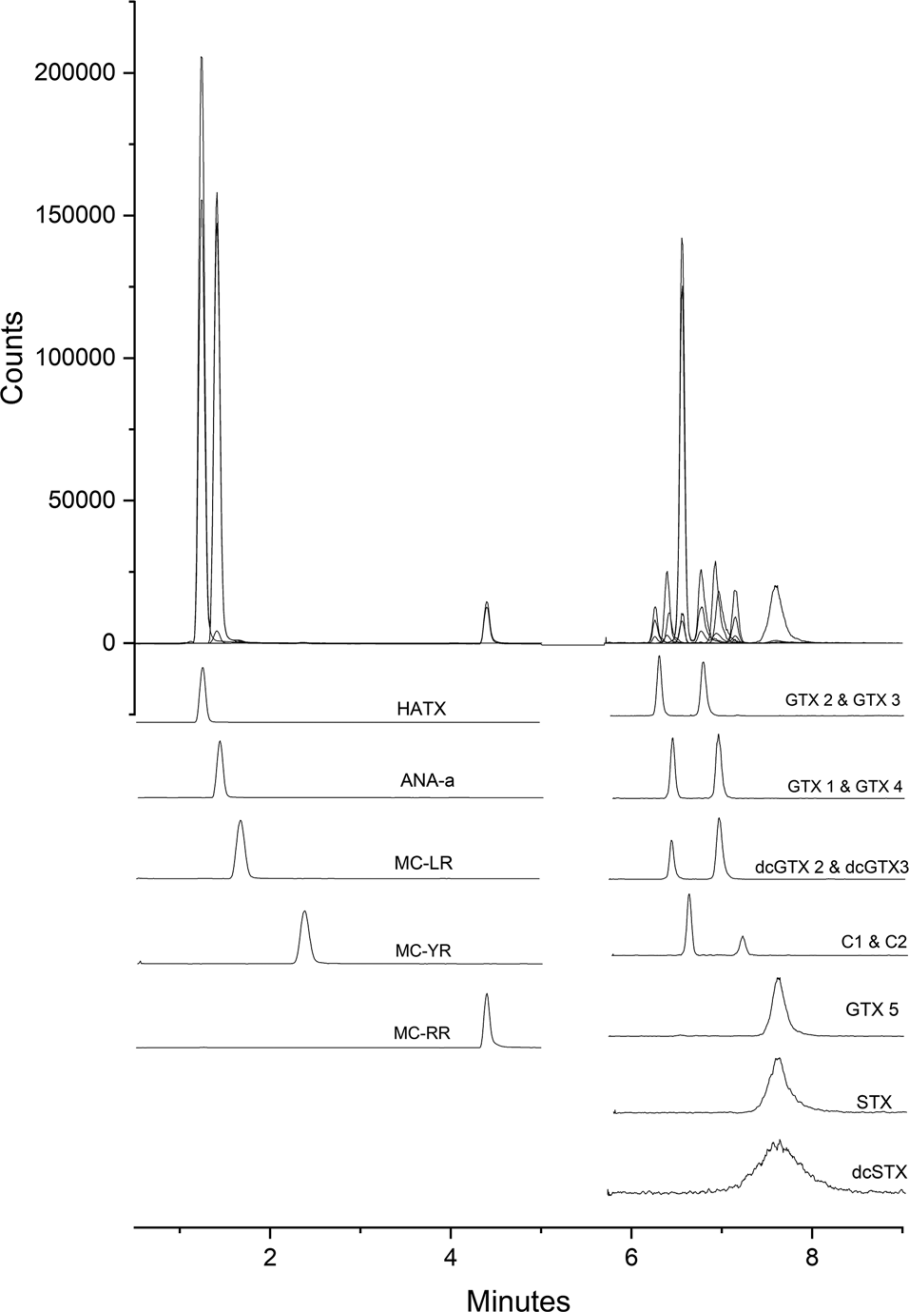
Optimized separation of the 16 analytes. The upper pane is a scaled total ion chromatogram, and separation of the analytes is shown in the lower panes.

Ammonium formate levels were tested at concentrations of 10, 7, 5, and 2 mM, with 2 mM providing baseline separation of the epimers. The optimal pH of the aqueous mobile phase was 3.5, with pH > 3.5 exhibiting a decrease in resolution and pH < 3.5 resulting in peak broadening. FA concentrations in the organic mobile phase of 0.1%, 0.25%, and 0.5% were examined, with 0.25% providing the best results. Finally, column temperatures between 20°C and 50°C were tested, with 40°C selected. A flow rate of 0.5 mL min^−1^ minimized peak broadening and increased resolution. The optimized separation is shown in Figure 2.

To date, there is only one published multiclass LC‐MS/MS method that includes ATXs, MCs, and STXs [33]. It used RPLC‐MS/MS to determine all the analytes included here except STX and dcSTX, which were not retained on the reverse‐phase column employed. They were unable to obtain resolution of the STX epimer pairs or obtain unique quantifier and qualifier MRM transitions needed for positive identification and quantification. Furthermore, no validation data was included [33]. In comparison, the method described here was able to separate the epimer pairs and obtain unique MRM transitions for their identification. Validation of the method is described in 3.5.

### 3.3 Multiclass Extraction of Algal Cyanotoxins

Extraction solvents for MCs and ATXs typically include a high concentration of organic solvent, whereas STXs require an acidic solution. Here we sought to simplify the process by acidifying an MC extraction solvent of 80:20 ACN:H_2_O. FA concentrations of 0%, 0.1%, 0.25%, and 0.5% were examined, with the MCs peak area highest at 0.1%, whereas the STXs had the lowest peak area at this concentration, differing from extraction methods in the literature [20, 26, 34, 35]. However, due to the considerably lower concentration of MCs detected compared to STXs in the algal sample extracts, a 0.1% acid concentration was selected. This choice aligned with available literature on STXs extraction [26, 36].

### 3.4 Method Validation

The LODs and LOQs for each of the analytes were between 0.00770 and 9.75 µg L^−1^ and 0.0257 and 32.5 µg L^−1^ respectively, with all cyanotoxins showing adequate linearity (*R* ^2^ values ≥ 0.991). The method exhibited sufficient repeatability, reported as a percentage relative standard deviation (%RSD), which was calculated from seven repeat injections of one standard to be 0–1.74 inter‐day for retention time and 4.54–27.6 inter‐day for peak area. The accuracy, determined via spike recovery, was appropriate with calculated percent recoveries of 75.6%–117%. All the analytical figures of merit are presented in Tables 1 and 2.

**TABLE 1:** Validation data, including LOD (µg L^−1^), LOQ (µg L^−1^), linearity (*R* ^2^), and linear range.

Toxin name	LOD	LOQ	*R* ^2^	Linear range
**HATX**	0.00770	0.0257	0.993	0.100–400 µg L^−1^
**ANA‐a**	0.0485	0.162	0.995	0.100–400 µg L^−1^
**MC‐LR**	0.221	0.736	0.998	0.563–205 µg L^−1^
**MC‐YR**	0.438	1.46	0.998	5.14–206 µg L^−1^
**MC‐RR**	0.111	0.369	0.998	0.548–199 µg L^−1^
**GTX 2**	5.33	17.8	0.993	38.8– 1550 µg L^−1^
**GTX 1**	9.75	32.5	0.991	52.8–2110 µg L^−1^
**dcGTX 2**	3.06	10.2	0.993	33.8–1350 µg L^−1^
**C1**	0.282	0.939	0.999	4.80–1740 µg L^−1^
**GTX 3**	0.0784	0.261	0.999	1.00–400 µg L^−1^
**GTX 4**	0.135	0.450	0.995	1.00–400 µg L^−1^
**dcGTX 3**	0.103	0.343	0.998	1.00–400 µg L^−1^
**C2**	0.145	0.484	0.997	1.00–400 µg L^−1^
**GTX 5**	0.110	0.368	0.997	1.00–400 µg L^−1^
**STX**	1.65	5.52	0.994	50.0–400 µg L^−1^
**dcSTX**	3.46	11.5	0.995	50.0–400 µg L^−1^

**TABLE 2:** Intra‐ and inter‐day precision, presented as %RSD, and the spiked recovery data for *Aphaniszomeno W*., where the matrix spike concentrations are presented within Table S2, determined for each analyte included within the method.

Toxin name	%RSD retention time		%RSD peak area		Matrix spike recovery (%)
	Intra‐day	Inter‐day	Intra‐day	Inter‐day	
**HATX**	0	0.427	3.66	24.1	100
**ANA‐a**	0.276	0.722	3.50	27.6	95.8
**MC‐LR**	0	0.767	3.20	7.65	88.4
**MC‐YR**	0.216	0.810	9.71	17.6	101
**MC‐RR**	0.115	0.223	4.66	4.54	97.7
**GTX 2**	0.0509	0.181	4.52	17.8	105
**GTX 1**	0.0732	0.221	6.79	18.2	75.6
**dcGTX 2**	0.149	0.266	16.4	21.5	99.0
**C1**	0.0300	0.0377	5.73	13.9	94.4
**GTX 3**	0.0826	0.248	5.25	11.4	113
**GTX 4**	0.130	0.284	6.64	13.9	82.0
**dcGTX 3**	0.150	0.218	5.93	15.1	95.8
**C2**	0.101	0.0920	4.05	9.47	104
**GTX 5**	0.137	0.399	2.84	8.08	103
**STX**	0.304	0.713	6.92	8.15	117
**dcSTX**	0.784	1.74	5.29	5.62	117

The multiclass method exhibited the highest sensitivity for the ATXs, with LODs below 0.050 µg L^−1^, and LOQs under 0.20 µg L^−1^. These results are superior to other HILIC‐MS/MS methods that measured ATXs, which had LODs for ANA‐a ranging from 4 [37] to 77.1 µg L^−1^ [26]. RPLC‐MS/MS is more commonly used for the analysis of ANA‐a and HATX and can achieve LODs and LOQs of 0.03, 0.10, 0.01, and 0.03 µg L^−1^, respectively [38]. The RPLC‐MS/MS values are comparable to the LODs and LOQs reported here, indicating the suitability of HILIC‐MS/MS for the analysis of ATXs.

The MCs had LODs and LOQs ranging from 0.111 to 0.438 µg L^−1^ and 0.369 to 1.46 µg L^−1^ respectively. Overall, the determined LODs and LOQs for MCs are low enough for the multiclass method to be employed as a screening method, where the regulated value is 1.3 µg L^−1^ for MCs in Australian drinking water [39]. An alternate HILIC‐MS/MS method that contained MCs had an LOD of 18 µg L^−1^ and an LOQ of 60 µg L^−1^ for MC‐LR and an LOD of 15 µg L^−1^ and an LOQ of 50 µg L^−1^ for MC‐RR [40]. Another HILIC‐MS/MS method had LODs and LOQs lower than what was presented here for MC‐LR, while ANA‐a and MC‐RR have comparable validation characteristics to what is presented within this study [41]. Similar to the ATXs, RPLC‐MS/MS is more commonly used, providing lower LODs and LOQs ranging from 0.13 to 0.30 µg L^−1^ and 0.40 to 0.90 µg L^−1^ respectively, and also obtaining lower inter‐day %RSDs for peak area and retention time [42]. The advantages of RPLC‐MS/MS cannot be matched with HILIC‐MS/MS; however, these results show that this method was fit for purpose.

The analysis of STXs using HILIC‐MS/MS is often complicated by shifting retention and fluctuating peak intensities [25]. The results in Table 2 show improved %RSDs for retention time and peak area for a broad range of STXs commonly analyzed [25]. The shifting retention and peak area, which was not observed here, is evidenced in the limits of analysis obtained by these other methods, with LODs ranging from 2.06 to 9.51 µg L^−1^ [20]. These values are all higher than the LODs shown in Table 1, with their lowest LOD of 2.06 µg L^−1^ for GTX 4 being 15 times higher than the LOD of 0.135 µg L^−1^ reported for this method. Other methods specific to the STX in paralytic shellfish poisoning, STX, GTX 1, and GTX 2, did manage to obtain lower LODs than those reported here; however they were within the same order of magnitude and required a 45 min run time for fewer analytes [45]. The use of negative ESI was shown to improve the LODs for GTX 1, GTX 2, dcGTX 2, STX, and dcSTX in a method that analyzed the same 11 STX; however, the improvements were modest [27]. Multiclass cyanotoxin HILIC‐MS/MS methods that contain fewer analytes have inferior LODs than those reported here. For example, one method had LODs of 35.8 µg L^−1^ for STX and 4 µg L^−1^ for ANA‐a [37], and another had LODs of 4.23 µg L^−1^ for dcGTX 3, 42.3 µg L^−1^ for dcGTX 2, 17.9 µg L^−1^ for STX, 20 µg L^−1^ for dcSTX, 47.4 µg L^−1^ for GTX 2, 4.74 µg L^−1^ for GTX 3, and 77.1 µg L^−1^ for ANA‐a [26]. This indicates that while HILIC‐MS/MS does allow for multiclass cyanotoxin analysis, creating a sensitive and robust method involves rigorous optimization and consideration of conditions to produce the most sensitive and repeatable method.

The accuracy of the analysis method was obtained via spiking an algal extract and determining the matrix spike recovery (%). It should be noted that here accuracy is only assessing matrix interferences and is not a measure of extraction recovery. The results are presented in Table 2 with adequate matrix spike recoveries for all analytes ranging from 75.6% to 117%.

### 3.5 Quantification of Cyanotoxins From Cyanobacteria Samples

**FIGURE 3:**
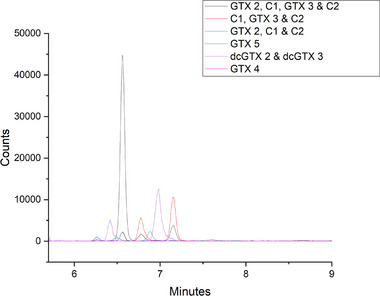
MRM chromatogram of *Dolichospermum circinale*, displayed with legend corresponding to individual MRM's.

A range of cyanobacterial cultures and cyanobacteria samples collected from the environment were tested using the optimized multiclass cyanotoxin extraction and analysis methods. The results are shown in Table S5. All MCs and 10 out of the 11 STXs included in the method were measured above the LOD from at least one culture. *Dolichospermum circinale* is a known producer of STXs, including the epimers GTX 1 and GTX 4 in select species [46, 47]. GTX 4 was detected and quantified here, with the lowest concentration of all the STXs in the sample, while GTX 1 was not detected (see Figure 3).

MCs were detected in the *Microcystis aeruginosa* species, with individual strains producing different MC congeners at varying concentrations. It is known that some *Microcystis* strains only produce MC‐LR, aligning with the results for the MASHOI‐AO5 strain [48], while there are other strains that produce multiple MCs, including MC‐LR, MC‐RR, and MC‐YR [49, 50]. The difference in concentrations between the two MASHOI‐AO5 culture strains could be due to a number of variables, such as the age of the cultures or variation in culture conditions, which can all affect the determined concentration [51].

## 4 Conclusion

We developed a multiclass HILIC‐MS/MS method for the detection and analysis of 11 STXs, 2 ATXs, and 3 MCs in a single chromatographic run. The method was validated, assessing the limits of analysis, linearity, inter‐ and intra‐day precision, and accuracy, and provided sufficient results to screen and quantify cyanotoxins in cyanobacterial samples. The optimized method was applied to 19 algal cultures and field samples and determined the presence of MCs and STXs. This comprehensive approach has the potential to contribute significantly to the ongoing efforts to monitor and ensure water safety while also expanding the knowledge on multiclass cyanotoxin analysis, as these three different classes have not been analyzed together using HILIC‐MS/MS.

## Author Contributions

**Rosemary Bergin**: investigation, validation, methodology, writing–original draft. **Siobhan Peters**: validation, methodology, writing–review and editing. **Simon Mitrovic**: conceptualization, resources, writing–review and editing. **David Bishop**: conceptualization, resources, writing–review and editing, supervision.

## Ethics Statement

The authors have nothing to report.

## Conflicts of Interest

The authors declare no conflicts of interest

## Supporting information



Supporting Information

## Data Availability

Data are available on request from the authors.
