# Supplementary material for: Simultaneous Multiclass Analysis of Cyanotoxins in Cyanobacterial Samples Using Hydrophilic Interaction Liquid Chromatography‐Tandem Mass Spectrometry
Source: J Sep Sci. 2025 Mar 19;48(3):e70121. doi: 10.1002/jssc.70121 (PMC11923511; doi:10.1002/jssc.70121)

**SUPPLEMENTARY SECTION:**

**Table 1:** Table displaying the Mobile phase gradient at different time points during the 15-minute run time, where the mobile phase compositions are given as percentages.

| **Time point (minutes)** | **% of mobile phase B** | **% of mobile phase A** |
| --- | --- | --- |
| **0.00** | 90% | 10% |
| **3.00** | 90% | 10% |
| **3.01** | 80% | 20% |
| **4.00** | 77% | 23% |
| **5.50** | 73% | 27% |
| **8.99** | 70% | 30% |
| **9.00** | 50% | 50% |
| **10.00** | 50% | 50% |
| **10.01** | 90% | 10% |
| **15.00** | 90% | 10% |

**Table** **2:** Table containing the MRM transitions for each analyte, including the analyte name, the precursor ion, the product ion, the collision energy, and the dwell time.

| Analyte name | Transition made from | Precursor mass | Product mass | Collision energy | Dwell time |
| --- | --- | --- | --- | --- | --- |
| MC-LR | [M+H]^+^ | 995.4 | 135.1* | 75 | 50 |
|  |  |  | 212.8 | 70 | 50 |
|  |  |  | 102.9 | 70 | 50 |
| MC-YR | [M+H]^+^ | 1046 | 134.8* | 60 | 50 |
|  |  |  | 213 | 60 | 50 |
|  |  |  | 106.6 | 80 | 50 |
| MC-RR | [M+2H]^2+^ | 520 | 135.2* | 30 | 50 |
|  |  |  | 103.2 | 70 | 50 |
|  |  |  | 127 | 50 | 50 |
| ANA-a | [M+H]^+^ | 166.1 | 91.1 | 30 | 10 |
|  |  |  | 130.8 | 15 | 10 |
|  |  |  | 148.8* | 12 | 10 |
| HATX | [M+H]^+^ | 180 | 57 | 20 | 20 |
|  |  |  | 91 | 18 | 20 |
|  |  |  | 163* | 11 | 20 |
| C^13^ ANA-a | [M+H]^+^ | 170.1 | 153.1* | 12 | 5 |
|  |  |  | 134.8 | 16 | 5 |
|  |  |  | 93.0 | 30 | 5 |
| STX | [M+H]^+^ | 300.1 | 109.9 | 46 | 5 |
|  |  |  | 138 | 30 | 5 |
|  |  |  | 204.1* | 23 | 5 |
| dcSTX | [M+H]^+^ | 257.1 | 125.8* | 20 | 5 |
|  |  |  | 137.9 | 30 | 5 |
|  |  |  | 239.1 | 15 | 5 |
| dcGTX 2 | [M+H-SO_3_]^+^ | 273.0 | 255.1 | 18 | 5 |
|  | [M+H]^+^ | 353 | 273* | 6 | 5 |
| dcGTX 3 | [M+H]^+^ | 353 | 255.1* | 18 | 5 |
|  |  |  | 335 | 9 | 5 |
| GTX 1 | [M+H-SO_3_]^+^ | 332 | 314* | 18 | 5 |
|  |  |  | 107.9 | 30 | 5 |
|  |  |  | 236 | 25 | 5 |
| GTX 2 | [M+H-SO_3_]^+^ | 316 | 147.6* | 23 | 5 |
|  |  |  | 220 | 26 | 5 |
| GTX 3 | [M+H]^+^ | 396 | 298.1* | 13 | 5 |
|  |  |  | 315.9 | 6 | 5 |
|  |  |  | 337.8 | 7 | 5 |
| GTX 4 | [M+H]^+^ | 412 | 314* | 15 | 5 |
|  |  |  | 394 | 6 | 5 |
|  |  |  | 332 | 12 | 5 |
| GTX 5 | [M+H]^+^ | 380 | 300* | 11 | 5 |
|  |  |  | 138 | 40 | 5 |
|  |  |  | 203.8 | 36 | 5 |
| C1 | [M+H-SO_3_]^+^ | 396.1 | 316.1* | 13 | 5 |
|  |  |  | 236.8 | 26 | 5 |
|  | [M+H]^+^ | 476 | 316.1 | 13 | 5 |
| C2 | [M+H]^+^ | 476 | 316.1 | 13 | 5 |
|  | [M+H-SO_3_]^+^ | 396.1 | 298.1* | 20 | 5 |
|  |  |  | 236.8 | 26 | 5 |

**Table 3:** Concentrations used when spiking algal samples (matrix spike recovery) and when determining %RSD in calibration curves constructed for analytes, when multiple analytes were included in one standard, the listed concentrations follow the same order as the names, where the concentration is in µg L^-1^.

| **Analyte name** | **Concentration used in matrix spikes (µg L^-1^)** |
| --- | --- |
| **MC-RR, MC-YR, and MC-LR** | 19.9, 20.6, 20.5 |
| **ANA-a** | 50 |
| **HATX** | 50 |
| **GTX 5** | 50 |
| **STX** | 50 |
| **dcGTX 2 and dcGTX 3** | 135, 50 |
| **GTX 1 and GX 4** | 211, 50 |
| **GTX 2 and GTX 3** | 155, 50 |
| **dcSTX** | 50 |
| **C1 and C2** | 174, 50 |

**Table 4.** The STX functional groups at the R positions designated in Supplementary Figure 1.

| **Toxins** | **R^1^** | **R^2^** | **R^3^** | **R^4^** |
| --- | --- | --- | --- | --- |
| **Saxitoxin (STX)** | H | H | H | Carbamoyl |
| **Gonyautoxin 1 (GTX 1)** | OH | H | OSO_3_^-^ | Carbamoyl |
| **Gonyautoxin 2 (GTX 2)** | H | H | OSO_3_^-^ | Carbamoyl |
| **Gonyautoxin 3 (GTX 3)** | H | OSO_3_^-^ | H | Carbamoyl |
| **Gonyautoxin 4 (GTX 4)** | OH | OSO_3_^-^ | H | Carbamoyl |
| **Gonyautoxin 5 (GTX 5)** | H | H | H | N-sulfocarbamoyl |
| **Decarbamoylsaxitoxin (dcSTX)** | H | H | H | Decarbamoyl |
| **Decarbamoylgonyautoxin-2 (dcGTX 2)** | H | H | OSO_3_^-^ | Decarbamoyl |
| **Decarbamoylgonyautoxin-3 (dcGTX 3)** | H | OSO_3_^-^ | H | Decarbamoyl |
| **N-sulfocarbamoylgonyautoxin-2 (C1)** | H | H | OSO_3_^-^ | N-sulfocarbamoyl |
| **N-sulfocarbamoylgonyautoxin-3 (C2)** | H | OSO_3_^-^ | H | N-sulfocarbamoyl |

**Table 5:** Table presenting the types of algae samples tested, the toxins identified and the concentration of the identified toxins in mg kg^-1^, where N.d. stands for not detected.

| **Algae samples tested** | **Toxins identified** | **Concentration of toxins (mg kg^-1^)** |
| --- | --- | --- |
| **Microcystis aeruginosa (MAG-LO2)** | MC-RR, MC-LR, MC-YR | 1.38×10^4^, 797, 3.80×10^3^ |
| **Microcystis aeruginosa 2 (MASHOI-A05)** | MC-LR | 1.37×10^5^ |
| **Microcystis aeruginosa 4 (MASHOI-A05)** | MC-LR | 6.39×10^4^ |
| **Leptolyngbya W.** | N.d. | N.d. |
| **Aphanocapsa W.** | N.d. | N.d. |
| **Planktothrix W.** | N.d. | N.d. |
| **Oscillatoria** | N.d. | N.d. |
| **Dolichospermum sp. 1** | N.d. | N.d. |
| **Dolichospermum sp. 2** | N.d. | N.d. |
| **Microcystis sp.** | N.d. | N.d. |
| **Microcystis sp. (D1956FD1236)** | N.d. | N.d. |
| **Microcystis sp. (F43b….D1)** | N.d. | N.d. |
| **Dolichospermum 1 (PF5 2a Fe4)** | N.d. | N.d. |
| **Dolichospermum straight chain GTD** | N.d. | N.d. |
| **Aphaniszomeno W.** | N.d. | N.d. |
| **Microcystis Flos-aqua** | N.d. | N.d. |
| **Anabaena sp.** | N.d. | N.d. |
| **Aged Leptolyngbia W.** | N.d. | N.d. |
| **Dolichospermum circinale** | C1, C2, GTX 2, GTX 3, GTX 4, GTX 5, dcGTX 2, dcGTX 3, dcSTX, STX | 1.96×10^5^, 6.50×10^4^, 1.63×10^4^, 1.73×10^4^, 128, 2.09×10^3^, 6.72×10^3^, 3.55×10^3^, 8.90×10^3^, 5.72×10^3^ |

**Figure 1:** The general structure of STXs, where R groups define the different STXs analogous.


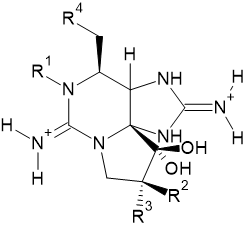


**Figure 2:** Product scan of dcGTX 2 and dcGTX 3.


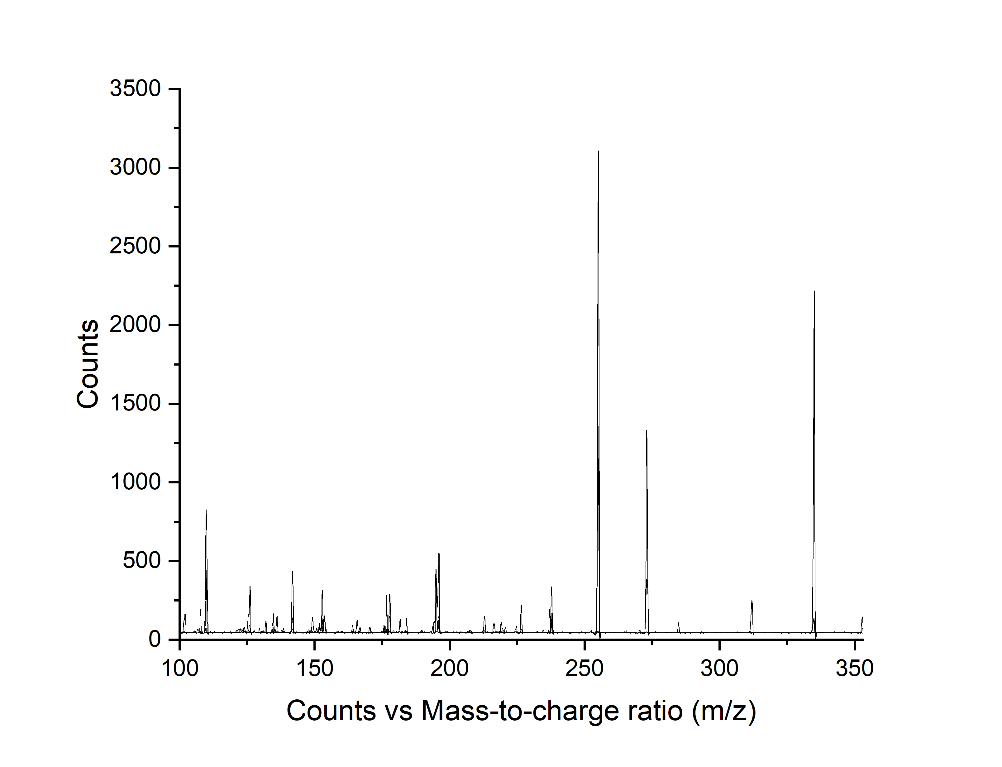


**Figure 3:** Product scan of GTX 2 and GTX 3.


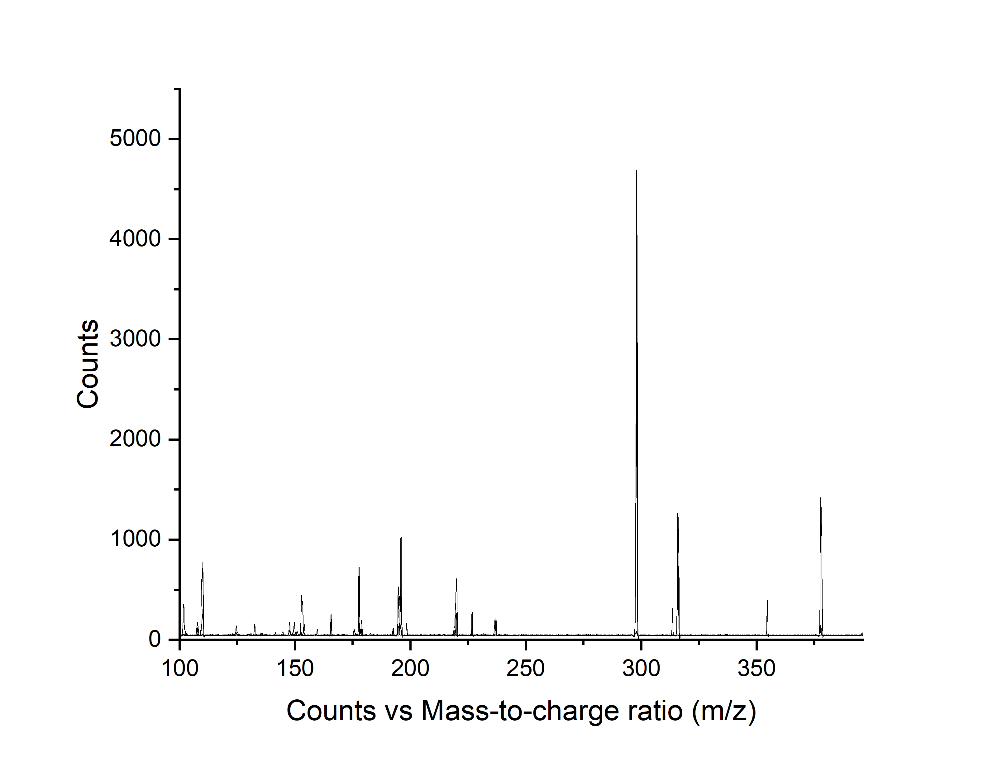


**Figure 4:** Product scan of C1 and C2.


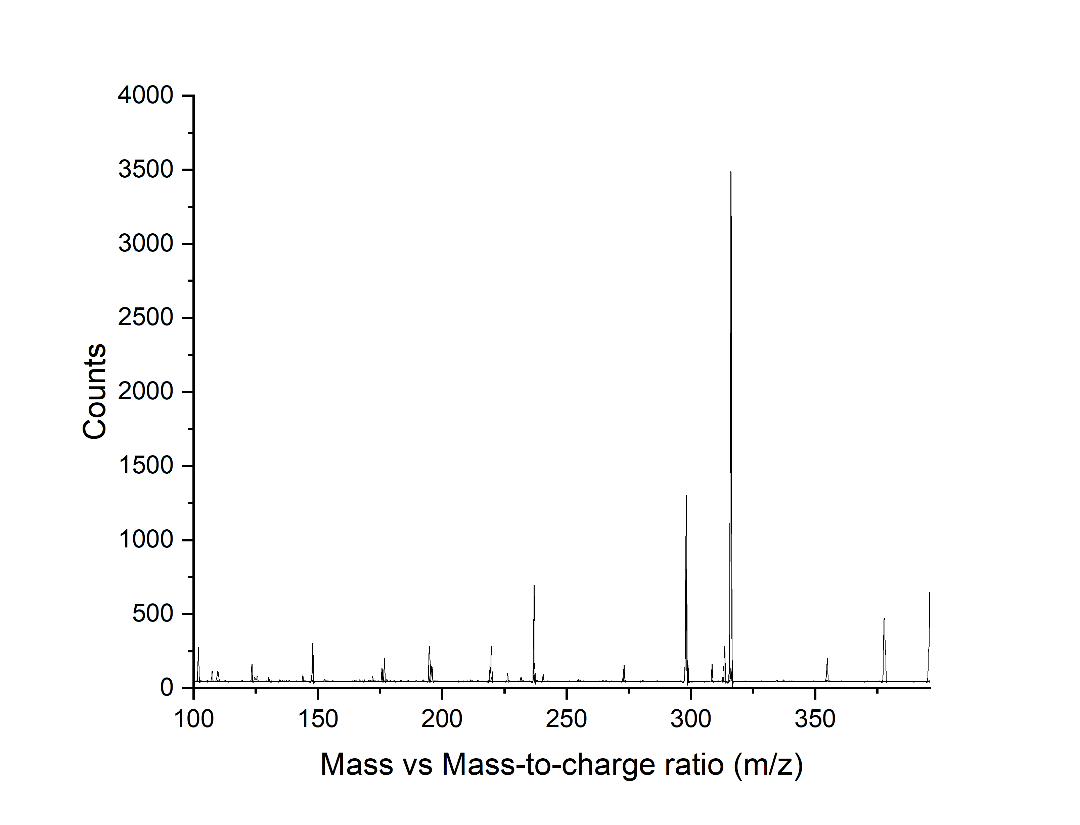

Supplement: Supplementary file 1 — Supporting Information [file JSSC-48-e70121-s001.docx]
